# Allergic Contact Dermatitis to Colophonium in a ‘Carnival Mask’

**DOI:** 10.1111/cod.70020

**Published:** 2025-08-28

**Authors:** Gabriela Blanchard, Olivier Sorg, Pierre Piletta‐Zanin

**Affiliations:** ^1^ Dermatology Department Geneva University Hospital Geneva Switzerland; ^2^ Clinical Pharmacology and Toxicology Unit University of Geneva Geneva Switzerland

**Keywords:** abietic acid, allergic contact dermatitis, CAS 514‐10‐3, CAS 8050‐09‐7, case report, colophonium, mask, rosin

Colophonium is a frequent cause of allergic contact dermatitis (ACD) with an estimated prevalence of 3.1% among patch‐tested patients in Europe [1]. Abietic acid isomers are the main components of colophonium, and their oxidation products are thought to be the primary allergens in colophonium [2].

## 1 Case Report

**FIGURE 1:**
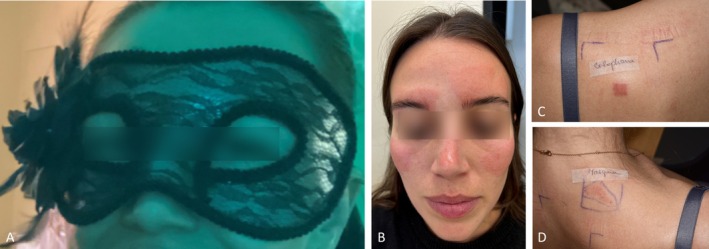
(A) The colophanium‐containing face mask worn by the patient. (B) Well‐demarcated facial erythema consistent with allergic contact dermatitis. (C) Strong positive patch test reaction to colophonium on day 4 (+++). (D) Strong positive patch test reaction to the patient's mask on day 4 (+++).

A 30‐year‐old Caucasian female developed a well‐demarcated, itchy facial erythema within 24 h of wearing a Halloween mask (Figure 1A,B), consistent with ACD. She was treated with topical corticosteroids followed by topical pimecrolimus, until reaching complete remission. Patch tests were performed using the European baseline, preservative and emulsifier series (supplied by Chemotechnique Diagnostics, Vellinge, Sweden) using IQ Ultra chambers (Chemotechnique Diagnostics) and the patient's own products, including a piece of the mask used (‘as is’). Allergens were applied for 48 h. Readings on day (D) 2 and D4 showed +++ positive reactions for colophonium (20% Pet) (Figure 1C), as well as the patient's mask (Figure 1D). This led us to hypothesise that the mask likely contained colophonium. As the individual mask ingredients were not available, we proceeded with a high‐performance liquid chromatography analysis to confirm this hypothesis. Abietic acid was found at a concentration of 2.65 ± 0.02 mg/g in both the frontal and temporal zones of the mask (see Supporting Information S1 and S2).

## 2 Discussion

The detection of abietic acid in both the frontal and temporal zones of the patient's mask confirms the diagnosis of ACD caused by colophonium derivatives. Colophonium is a naturally occurring resin found in the sap of coniferous trees. It is commonly used as a component of adhesives, such as tapes and glues [3], as well as in cosmetics, paper products and footwear [4]. It is interesting to note that the interior part of the mask (in contact with the patient's skin) was smooth without any adhesivity. According to Regulation No. 1272/2008 of the European Parliament and Council, the manufacturers and importers of colophony‐containing products should label them ‘*Contains colophony. May produce an allergic reaction*’ if the colophony content exceeds 0.1% [5]. Nevertheless, due to the lack of standardised quantification methods, even in the absence of such labelling, clinicians should maintain a high degree of suspicion for sensitization to abietic acid or colophonium in cases of ACD, including those involving non‐adhesive products.

## Consent

The authors obtained written consent from the patient for their photographs and medical information to be published in print and online and with the understanding that this information may be publicly available. Patient consent forms were not provided to the journal but are retained by the authors.

## Conflicts of Interest

The authors declare no conflicts of interest.

## Supporting information


**Data S1:** Supporting Information.

**Figure S1:** Chromatogram for abietic acid.

## Data Availability

Data sharing is not applicable to this article as no new data were created or analysed in this study.
