# Supplementary material for: Allergic Contact Dermatitis to Colophonium in a ‘Carnival Mask’
Source: Contact Dermatitis. 2025 Aug 28;93(6):533–4. doi: 10.1111/cod.70020 (PMC12586276; doi:10.1111/cod.70020)
Supplement: Supplementary file 1 — Data S1: Supporting Information. [file COD-93-533-s001.docx]

**Supplementary Files for " Allergic contact dermatitis to colophonium in a “Carnival mask” "**

by *Gabriela Blanchard^1^, Olivier Sorg^2^ and Pierre Piletta-Zanin^1^*

^1^ Dermatology Department, Geneva University Hospital, Geneva, Switzerland

^2^ Clinical Pharmacology and Toxicology Unit, University of Geneva, Geneva, Switzerland

Method : HPLC analysis of parts of the mask

Rectangles (≈ 1.5 g) from the frontal and temporal zones of the patient’s mask were minced, grounded, and extracted with 10 ml ethanol overnight at 4°C, then centrifuged (1'500 g/10 min). Supernatants were diluted 1:100 with 0.2% acid acetic in acetonitrile. Pellets were re-extracted with 8 ml acetone overnight at 4°C and centrifuged as above. The supernatants were evaporated to dryness under nitrogen flux and reconstituted with 2 ml ethanol and diluted 1:10 with 0.2% acetic acid in acetonitrile. Samples were analysed by HPLC (injection volume of 100 µL) using a C_18_ NUCLEODUR Pyramid 3 µm column (4.6 x 250 mm, Macherey-Nagel, Oensingen, Switzerland) eluted with 0.2% acetic acid in acetonitrile in isocratic conditions. The mobile phase was pumped with an Agilent 1260 quaternary pump at a flow rate of 0.8 mL/min and monitored at 240 nm with an Agilent 1100 Series diode-array detector (Agilent Technologies, Basel, Switzerland). Pure abietic acid showed a maximal absorption peak at 240 nm and a retention time of 7.52 min. Abietic acid was identified in all samples with their retention time and their absorption spectrum by comparison with the analytical standard (Figure S1). For each of the two zones of the mask, the amount of abietic acid was obtained by the sum of the two extractions. The concentrations of abietic acid in the frontal and temporal zones of the mask were 2.63 mg/g and 2.67 mg/g, respectively.

Figure S1. Chromatogram for abietic acid


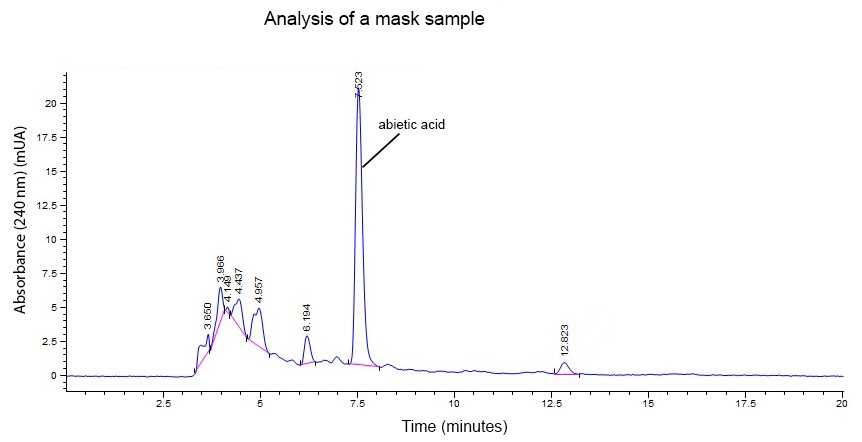


High-performance liquid chromatography confirms the presence of abietic acid, component of colophonium, in the patient’s mask. Pure abietic acid showed a maximal absorption peak at 240 nm and a retention time of 7.52 min.
