# Supplementary figures and images for: Allergic Contact Dermatitis to Colophonium in a ‘Carnival Mask’
Source: Contact Dermatitis. 2025 Aug 28;93(6):533–4. doi: 10.1111/cod.70020 (PMC12586276; doi:10.1111/cod.70020)

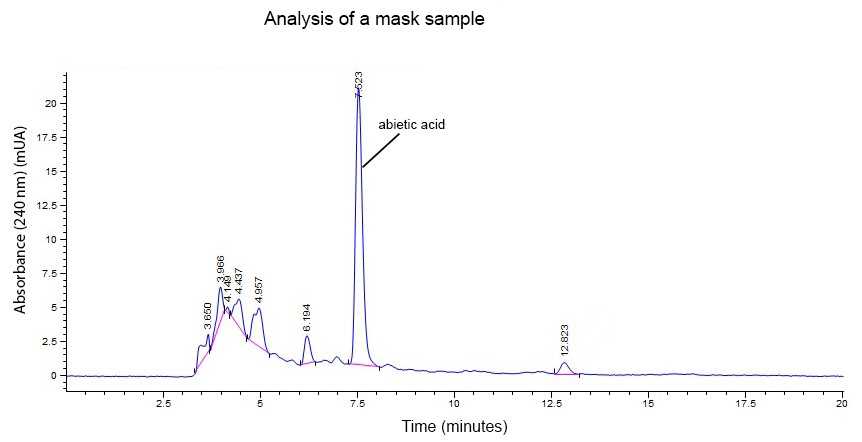

Supplement: Supplementary file 2 — Figure S1: Chromatogram for abietic acid. [file COD-93-533-s002.jpg]
